# The Potential Misuse of DNA Probe for the Detection of *Neisseria gonorrhoeae* and *Chlamydía trachomatis* When Used for Test of Cure

**DOI:** 10.1155/S1064744997000720

**Published:** 1997

**Authors:** Gilles R. G. Monif

**Affiliations:** Department of Obstetrics and Gynecology Creighton University School of Medicine Omaha NE USA


Infectious Diseases in Obstetrics and Gynecology 5:395–396 (1997)
© 1998 Wiley-Liss, Inc.

### The Potential Misuse of DNA Probe for the Detection of *Neisseria gonorrhoeae* and *Chlamydía trachomatis* When Used for Test of Cure

**Gilles R.G. Monif, M.D.**
*Department of Obstetrics and Gynecology, Creighton University School of Medicine, Omaha, NE*

The use of cell culture systems for the detection of *Chlamydia trachomatis* is the gold standard.[1] However, the sensitivity of a single endocervical specimen may be only 70–90.[2–3] Unfortunately, cell culture is labor intensive and requires 48 hours for completion. These problems fostered the development of simplified rapid diagnostic tests which bypassed the issue of organism viability. The first detection systems were based on either immunodetection of solubilized chlamydia antigens (enzyme immunoassay) or direct visualization using chlamydia-specific fluorescin-conjugated monoclonal antibodies (direct fluorescent antibody test). The relative sensitivity and poorer positive predictive values of these methods led ultimately to their relative abandonment in favor of tests based on DNA/rRNA hybridization.[4–5]

The ability of the DNA probe to detect both *Neisseria gonorrhoeae* and *C. trachomatis* in a single test has made nucleic acid probe testing an attractive alternative. The long-term cost of false-negative gonococcal cultures outweighed the cost differential between properly handled specific cultures for *N. gonorrhoeae* and *C. trachomatis* and a DNA probe (Table 1).

### Test of Cure

With the recent introduction of single-dose therapy for both *N. gonorrhoeae* and *C. trachomatis*, routine test of cure during the immediate post-therapy period is not recommended by the Centers for Disease Control. The CDC recommends that test of cure for *N. gonorrhoeae* or *C. trachomatis* be performed 7–14 days after completion of therapy if symptoms persist. While this strategy is probably valid for men, its applicability to asymptomatic women is not well documented. In the absence of symptomology, why should one do a test of cure? If a macrolide other than azithromycin has been utilized, the probability of poor compliance is significant. When compliance is questioned, a test of cure is advocated. Seventy percent of treatment failures with erythromycin appear to be related to compliance problems. Published cure rates with macrolides, such as erythromycin, are not totally comparable to those observed with doxycycline. A test of cure may be warranted three weeks after completion of treatment with these antibiotics. With non-cultured tests, residual chlamydial antigen and nucleic acids, in the absence of viable organisms, may result in a positive test. Such a positive result can be misinterpreted as a treatment failure. False-positive tests due to antigen persistence can occur up to three weeks after doxycycline therapy.

A second situation which appears to warrant a test of cure is that of an individual who is at high risk for reinfection. High-risk women should be rescreened one or two months after testing. By so doing, both therapeutic failures and bases of reinfection can be identified. Should the culture for *N. gonorrhoeae* be positive, one has the isolate for antibiotic-sensitivity studies. In the case of *C. trachomatis*, the use of culture precludes antigens from dead organisms causing a false-positive test.

Since the prevalence of infection in test of cure is very low, tests with the highest positive predictive value, e.g., culture when testing performed less than three weeks after therapy, are advocated. After three weeks, nucleic acid amplification tests such as polymerase chain reaction (PCR) and ligase chain reaction (LCR), which have high positive predictive values, can be used.

**TABLE 1.** Cost of current screening test for *Neisseria gonorrhoeae* (NG) and *Chlamydia trachomatis* (CT)

| | | |
|---|---|---|
| Cost of culture for NG* | $18.50 | Combined cost |
| Cost of culture for CT* | $57.95 | $76.45 |
| Cost of DNA probe for NG/CT* | $88.00 | |

*Creighton University/Saint Joseph Hospital

**TABLE 2.** Cost comparison of test of cure when either *N. gonorrhoeae* (NG) or *C. trachomatis* (CT) is identified

| Test | Cost | Differences |
|---|---|---|
| DNA probe NG/CT | $88.00 | — |
| Culture for CT | $57.95 | $30.05 |
| Culture for NG | $18.50 | $61.50 |

*Creighton University/Saint Joseph Hospital

**TABLE 3.** Test systems selection for identification of *C. trachomatis*

| Clinical situation | Test procedure of choice |
|---|---|
| Low-prevalence screening | DNA hybridization (ultimately amplified DNA probe technologies, polymerase chain reaction, LCR) |
| Sexual assault/abuse | Culture-only method recommended* |
| Test of cure | |
| Less than 3 weeks after therapy | Culture |
| Greater than 3 weeks after surgery | rRNA or DNA hybridization probes |

*If culture is obtained within 48 hours after exposure, it is recommended that a second culture be obtained in two weeks in the absence of preventive therapy.

### Use of Tests in the Context of Test of Cure

A recent survey of local practicing obstetric and gynecologic clinicians has shown that the nucleic acid probes are being used for test of cure. This is an uneconomical use of technology (Table 2). The cost of the DNA probe for both *N. gonorrhoeae* and *C. trachomatis* at Creighton University/Saint Joseph Hospital is $88.00. According to the CDC, the public health price for amplification tests is below $15.00, but this price is not available in the usual practice situation. The use of DNA probe is predicated on the superior sensitivity of this antigen-based technique over culture recovery of environmentally viable *N. gonorrhoeae*. Once the initial screening test is positive for one isolate, cost-efficacy dictates that the test of cure should be culture for that organism unless the ability to do so properly is lacking. A properly obtained culture for *N. gonorrhoeae* entails culture media incubated prior to plating, availability of ambient $CO_2$, and minimal delay between sampling and incubation. The test systems best suited for specific indications for *C. trachomatis* are listed in Table 3. Use of DNA probe as a test of cure is warranted only if both organisms were initially identified or no other means of documenting eradication is available.

### REFERENCES

1. Yang LI, Panke ES, Leist PA, et al: Detection of *Chlamydia trachomatis* endocervical infection in asymptomatic and symptomatic women: Comparison of deoxyribonucleic acid probe test with tissue culture. Am J Obstet Gynecol 65:1444–1453, 1991.
2. Lefebevre J, Leperriere H, Rousseau H, Masse R: Comparison of three techniques for detection of *Chlamydia trachomatis* in endocervical specimens for asymptomatic women. J Clin Microbiol 26:726–731, 1988.
3. LeBar W, Herschman B, Jemal C, Pierzchala J: Comparison of DNA probe, monoclonal antibody enzyme immunoassay and cell culture for the detection of *Chlamydia trachomatis*. J Clin Microbiol 27:826–828, 1989.
4. Blanding J, Hirsch L, Stranton N, et al.: Comparison of the Clearview Chlamydia, the PACE 2 assay, and culture for detection of *Chlamydia trachomatis* from cervical specimens in a low-prevalence population. J Clin Microbiol 31:1622–1625, 1993.
5. Clarke L, Sierra M, Daidone B, Lopez N, Covino JM, McCormack WM: Comparison of the Syva micro-track enzyme immunoassay and gen-probe PACE 2 with cell culture for diagnosis of cervical *Chlamydia trachomatis* infection in a high-prevalence female population. J Clin Microbiol 31:968–971, 1993.
